# E-cigarette exposure causes early pro-atherogenic changes in an inducible murine model of atherosclerosis

**DOI:** 10.3389/ftox.2023.1244596

**Published:** 2023-12-18

**Authors:** Bayan Alakhtar, Cynthia Guilbert, Nivetha Subramaniam, Vincenza Caruana, Kiran Makhani, Carolyn J. Baglole, Koren K. Mann

**Affiliations:** ^1^ Division of Experimental Medicine, Department of Medicine, McGill University, Montreal, QC, Canada; ^2^ Lady Davis Institute for Medical Research, Jewish General Hospital, Montreal, QC, Canada; ^3^ Research Institute of the McGill University Health Centre, Montreal, QC, Canada; ^4^ Department of Pharmacology and Therapeutics, McGill University, Montreal, QC, Canada; ^5^ Department of Pathology, McGill University, Montreal, QC, Canada

**Keywords:** e-cigarette, atherosclerosis, vaping, mouse model, hyperlipidaemia

## Abstract

**Introduction:** Evidence suggests that e-cigarette use (vaping) increases cardiovascular disease risk, but decades are needed before people who vape would develop pathology. Thus, murine models of atherosclerosis can be utilized as tools to understand disease susceptibility, risk and pathogenesis. Moreover, there is a poor understanding of how risk factors for atherosclerosis (i.e., hyperlipidemia, high-fat diet) intersect with vaping to promote disease risk. Herein, we evaluated whether there was early evidence of atherosclerosis in an inducible hyperlipidemic mouse exposed to aerosol from commercial pod-style devices and e-liquid.

**Methods:** Mice were injected with adeno-associated virus containing the human protein convertase subtilisin/kexin type 9 (PCSK9) variant to promote hyperlipidemia. These mice were fed a high-fat diet and exposed to room air or aerosol derived from JUUL pods containing polyethylene glycol/vegetable glycerin (PG/VG) or 5% nicotine with mango flavoring for 4 weeks; this timepoint was utilized to assess markers of atherosclerosis that may occur prior to the development of atherosclerotic plaques.

**Results:** These data show that various parameters including weight, circulating lipoprotein/glucose levels, and splenic immune cells were significantly affected by exposure to PG/VG and/or nicotine-containing aerosols.

**Discussion:** Not only can this mouse model be utilized for chronic vaping studies to assess the vascular pathology but these data support that vaping is not risk-free and may increase CVD outcomes later in life.

## 1 Introduction

Electronic cigarettes (e-cigarettes) made their market debut across the United States in 2006, promoted as a new and convenient alternative to tobacco smoking and advertised as a safer, non-carcinogenic option (Cahn and Siegel, 2011; Fadus et al., 2019). E-cigarette usage has surged to include non-smoking teens and young adults (Caponnetto et al., 2012; Civiletto and Hutchison, 2019; Hammond et al., 2019). A recent survey in Canada indicated that 59.3% of those aged 16–25 had utilized a pod-style e-cigarette cartridge within the past 6 months (Ahmad et al., 2022). A survey from 2017 indicated that of individuals aged 15–24, 19% were regular users of the JUUL brand of e-cigarette (Willett et al., 2019). JUUL was introduced in 2015 (Fadus et al., 2019) and at one point, was the most popular e-cigarette brand in North America dominating with a 70% market share (King et al., 2018; Jackler and Ramamurthi, 2019). Despite the dramatic increase in e-cigarette use among teens and young adults in the US and Canada (Civiletto and Hutchison, 2019), long-term health consequences of use are unknown.

E-cigarettes consist of a rechargeable battery, atomiser (heating coil), and e-liquid cartridge. E-liquids contain a solvent—usually propylene glycol (PG) and vegetable glycerin (VG)—nicotine, and various flavour additives (Traboulsi et al., 2020). While many of the components in e-liquids are deemed safe for oral consumption and thus, labelled “Generally Recognized as Safe” by the United States Food and Drug Administration, there is limited information about the safety of the inhaled aerosolised e-liquid (Kaur et al., 2018). JUUL, a pod style e-cigarette, contains the same amount of nicotine as two packs of cigarettes (Goniewicz et al., 2019). JUUL e-liquids contain a nicotine base and a weak organic acid which form a nicotine salt. This form of nicotine is less abrasive for the lungs and facilitates the delivery of higher amounts of nicotine compared to free-base nicotine (Pankow et al., 2017; Rao et al., 2020). Moreover, in JUUL devices, the heating coil is usually made of nichrome, a combination of nickel, chromium, and other metals. Potential leaching of toxic metals from these heated coils into e-liquid and vaping aerosols represents a public health concern (Traboulsi et al., 2020).

E-cigarette use may correlate with increased risk of cardiovascular disease (CVD), such as stroke and acute myocardial infarct (AMI) (Vindhyal et al., 2019; Zhao et al., 2020), although it may take decades to have sufficient human data. An underlying cause of CVD is atherosclerosis, the gradual development of fibro-fatty plaque in large arteries. Plaque development is triggered by dyslipidemia, which is characterised by increased low-density lipoprotein (LDL) levels and decreased high density lipoprotein (HDL). Mechanistically, circulating reactive oxygen species (ROS) generate oxidised lipids that can activate cells of the endothelial layer lining the arteries, increasing adhesion molecule expression (Falk, 2006). Circulating levels of these adhesion molecules are biomarkers for CVD (Hwang et al., 1997). These activated endothelial cells express adhesion molecules that firmly bind to circulating monocytes, resulting in their migration into the subendothelial space where they differentiate into macrophages (Falk, 2006). Macrophages subsequently become activated and scavenge the oxidised lipoprotein particles, triggering their transformation into foam cells that accumulate as a component of plaque. Lastly, vascular smooth muscle cells then proliferate to form a stabilised fibrous cap over the plaque protecting it from rupture (Falk, 2006).

Because atherosclerosis takes years to develop into symptomatic disease in humans, it becomes necessary to develop models of vaping-induced vascular disease with which to assess the risk of vaping. Although several studies have utilized the well characterized apolipoprotein E knockout mice (ApoE^−/−^), the use of ApoE^−/−^ mice limits the experimental design because the timing of hyperlipidemia cannot be varied. Moreover, these mice are always hyperlipidemic, and thus cannot be used to model important variables of exposure that occur in humans, such as adult-onset hyperlipidemia after adolescent vaping, for example,. Therefore, we utilized an inducible hyperlipidemic mouse model to study the vascular and immune effects of vaping. In this model, standard in-bred mouse strains infected with adeno-associated viral particles containing the human protein convertase subtilisin/kexin type 9 (AAV-PCSK9^DY^) variant develop hyperlipidemia due to increased recycling of LDL-R from the liver and subsequently will develop atherosclerosis (Roche-Molina et al., 2015; Goettsch et al., 2016). We then combined this inducible hyperlipidemic model with our published vaping regimen with JUUL e-cigarettes that mimics human puff topography (i.e., puff volume and puff interval) (Been et al., 2022) utilizing a 4-week exposure to either room air, the vehicle (PG/VG) or mango-flavored e-liquid containing 59 mg/mL nicotine. While 4 weeks is too premature for significant atherosclerotic plaque development in mice, we observed that there were early pro-atherogenic changes, suggesting that the AAV-PCSK9^DY^ model is appropriate for assessing the risk of atherosclerosis from long-term vaping.

## 2 Materials and methods

### 2.1 JUUL products

For all exposures, commercial JUUL products were utilised. The vehicle consisted of a 30:70 ratio of PG and VG purchased from Fusion Flavours (fusionflavours.ca). JUUL products containing 59 mg/mL nicotine were purchased from a local vape store.

### 2.2 Animal exposures

**FIGURE 1:**
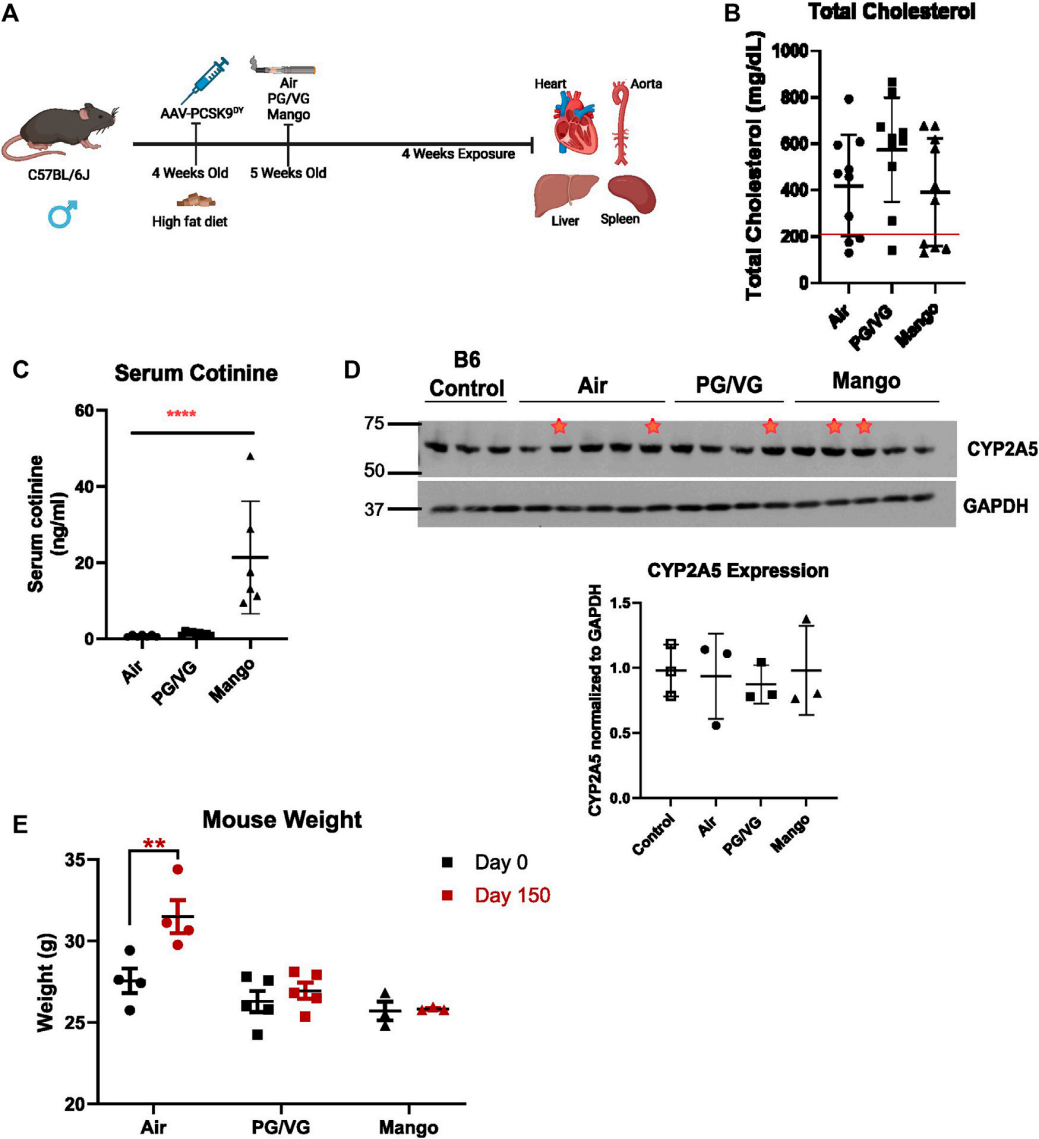
Experimental design and mouse model of hyperlipidemia. **(A)** C57BL/6J male mice were injected with AAV-PCSK9^DY^ virus and started on high fat diet on the same day. 1 week following injection mice were exposed to either room air, PG/VG only, or JUUL aerosol with mango flavour and 59 mg/mL nicotine. Mice were exposed to 4 puffs per min for 20 min 3 times a day for 4 weeks. Created with BioRender.com **(B)** Serum cholesterol level where a red line indicates 200 mg/dL total cholesterol level, below which mice were excluded. *n* = 10 **(C)** Serum cotinine level was measured by ELISA after 24 h of last exposure in hyperlipidemic mice. There was a significant increase in cotinine level in the JUUL mango group compared to Air and PG/VG groups (**** = p < 0.0001; *n* = 5). **(D)** CYP2A5 western blot comparing uninfected, unexposed C57BL/6J mice and AAV-PCSK9^DY^ mice exposed to air, PG/VG, or JUUL mango shows no difference among exposure groups. Red stars indicate those mice infected with AAV-PCSK9^DY^, but without hyperlipidemia (not included in densitometry). Densitometric data includes only those mice with cholesterol >200 mg/dL. Results are expressed as the mean ± SD. *n* = 3 **(E)** Body weight was measured before and after the exposure period. ** = p = 0.005 *n* = 3–5.

All procedures involving mice were approved by the McGill University Animal Care Committee (Protocol number 2013–7421) and carried out in accordance with the Canadian Council on Animal Care Committee. C57BL/6J mice were purchased from the Jackson Laboratory and bred in-house. Four-week-old male mice were injected intraperitoneally with 3 × 10^10^ AAV-PCSK9^DY^ viral particles (generated by GeneCopoeia, Inc. using plasmid from Addgene, pAAV/D377Y-mPCSK9, Cat# 58376) (Figure 1A). Male mice (10 mice/group) were utilized for optimization because the literature reports that female mice require more viral particles to generate the same level of atherosclerosis (Vozenilek et al., 2018). On the same day as injection, mice were started on a high-fat diet (ENVIGO high fat diet, TD.10825). One week following the injection, mice were randomly allocated to one of three groups: air, vehicle (PG/VG) or mango-flavored JUUL e-liquid. Air-exposed mice were placed within the device and received only room air. Exposures were performed using the SCIREQ^®^ inExpose™ system equipped with an extension for JUUL and a nose-only exposure module. Exposure parameters and puff profile were programmed using the flexiWare software. Mice were exposed to a puff regime consisting of three 20-min exposures per day for 4 weeks. The puff regime was 4 puffs per minute with a 78 mL puff volume, 2.4 s puff duration, and 3 h between exposure sessions as we recently published (Been et al., 2022). These usage parameters are consistent with human use patterns of e-cigarettes (Behar et al., 2015; Leavens et al., 2019; Vogel et al., 2019). To establish a baseline for the hyperlipidemic mice, wild-type C57BL/6J mice exposed to air only served as a negative control. Following the 4 weeks of exposure, mice were euthanized with isoflurane 1 day following the final exposure, and the aorta, heart, spleen, liver, and kidneys were dissected and collected. Increased lipid levels are a blood biomarker for adequate AAV-PCSK9^DY^ expression and subsequent LDL-R knock-down. Hypercholesterolemia (with total cholesterol levels > 200 mg/dL) reflects appropriate AAV-PCSK9^DY^ function (Maxwell and Breslow, 2004). Thus, plasma lipid levels were evaluated and only those mice with total cholesterol levels higher than 200 mg/dL (Figure 1B) were further analyzed as reflection of appropriate AAV-PCSK9^DY^ delivery.

### 2.3 Assessment of atherosclerotic lesions

Atherosclerotic lesions and lipid content were evaluated within the heart aortic sinus. The heart was rinsed, fixed, incubated in sucrose, and embedded in OCT. Frozen hearts were processed as previously described (Lemaire et al., 2011). Cryosections (7 µm) were generated from the aortic base throughout the aortic sinus, such that consecutive sections were placed on 10 different slides. Thus, individual slides contained between 7 and 10 sections. Slides were then stained with oil red O (Electron Microscopy Sciences) to visualize the plaque areas and were analyzed for their lipid content using ImageJ (National Institutes of Health).

### 2.4 Immunohistochemistry (IHC) staining

Vascular cell adhesion molecule-1 (VCAM-1) expression on the endothelial walls of the carotid and brachiocephalic artery (BCA) was evaluated using IHC staining. Arteries were dissected, rinsed, fixed in 4% paraformaldehyde, embedded longitudinally in paraffin, and processed. Then, consecutive 4 μ m ring sections of the arteries were sectioned. Paraffin-embedded arterial sections were deparaffinized and then washed under running tap water for 5 min and MQ H_2_O for 2 min. Antigen retrieval was performed using antigen retrieval solution (1X TRIS/EDTA pH 9 and 0.025% Triton X-100) in a pressure cooker for 20 min. After, slides were washed with wash buffer (TBS plus 0.025% Triton X-100). Slides were then incubated in 4.5% H_2_O_2_ in 24 mM NaOH for 30 min and then rinsed with the wash buffer. Then, slides were blocked in 10% goat serum (Jackson Immuno Research) with 1% BSA in TBS for 30 min and washed once. After, slides were incubated in Fc block plus mouse HRP (Cytiva) (1:100) for 30 min followed by incubation with the primary antibody against VCAM-1 (1:400; Abcam; cat # ab134047) diluted in TBS with 1% BSA at room temperature for 30 min. After, slides were rinsed with wash buffer and incubated with the corresponding secondary antibody (Envision + HRP-anti rabbit; DAKO; cat #K4003) for 1 h then washed 4 times with wash buffer. Next, a short incubation of 1 min with DAB substrate was used to visualize the VCAM-1 staining, followed by 20 s of hematoxylin to counterstain. Finally, tissues were dehydrated then coverslips mounted using Permount mounting media. Slides were visualized using automated digital microscopy system (AxioScan slide scanner) and analyzed using QuPath-0.2.3 by measuring the mean DAB Optical Density exclusively in the ECs.

### 2.5 Measurement of endothelial cell activation markers

Blood ( ≈ 0.4 mL) was drawn by cardiac puncture and plasma was isolated using EDTA-coated tubes. Intracellular cell adhesion molecule-1 (ICAM-1), platelet endothelial cell adhesion molecule (PECAM-1), endothelium-selectin (E-selectin), platelet-selectin (P-selectin), matrix metalloproteinase-9 (MMP-9) and plasminogen activator inhibitor 1 (PAI-1) levels were assessed using an immunoassay kit (multiplex bead-based, mouse Cardiovascular Disease Panel 1 7-Plex Discovery Assay^®^ Array (MDCVD1; Eve technology, Calgary, AB. Canada).

### 2.6 Measurement of total cholesterol and LDL levels

Total cholesterol, LDL, HDL, triglyceride, and glucose were assessed in plasma samples using the Bio-Rad Liquid Assayed Multiqual kit (The Centre for Phenogenomics, Toronto, Canada).

### 2.7 Measurement of cotinine levels

Serum cotinine levels were measured in plasma samples using an ELISA (Origene) as per the manufacturer’s instructions.

### 2.8 CYP2A5 western blot

Hepatic CYP2A5 levels were investigated using western blot. Frozen liver samples were ground to powder using liquid nitrogen, then incubated for 30 min in RIPA lysis buffer (BioBasic, Ontario, Canada) containing PhosStop and cOmplete, EDTA-free Protease inhibitor Complex (Sigma-Aldrich Canada Co. Ontario Canada). After preclearing the lysates with centrifugation for 20 min at 13,000 rpm, the amount of protein in each tissue lysate was quantified using a Bradford assay (Bio Rad). Then, an equal amount of protein (25 µg) from each sample was loaded and electrophoresed through a 7.5% SDS-polyacrylamide gel overnight. Proteins were then transferred to a PVDF membrane and blocked for 1 h with 5% fat free milk in Tris-buffered saline with Tween-20 (TBST). Membranes were then incubated with human CYP2A6 primary antibody, which cross-reacts with murine CYP2A5 (1:500) (Proteintech; cat #21721-1-AP) at 4°C overnight, and rabbit anti-GAPDH (1:3000; used as a control loading protein, Cell Signaling Technologies; cat #2118S). Membranes were washed three times with TBST for 10 min each prior to incubation with the secondary antibody (HRP-conjugated anti-rabbit; Cytiva; cat #NA934V) (1:3000) for 2 h at room temperature followed by washing three times with TBST for 10 min each. Signals were detected using prime ECL plus (Cytiva) and the membrane was exposed to an X-ray film (Thermo Scientific). Quantification and analysis of the expression of CYP2A5 and GAPDH were performed using ImageJ software.

### 2.9 Flow cytometry

The spleen was dissected and placed in a 1.5 mL Eppendorf filled with cold PBS. A single cell suspension was obtained by gentle pressure-dissociation of spleen in PBS using a pestle, and then passed through a 70-µm sterile cell strainer. Spleen cells were centrifuged 10 min at 300 × *g*, 4°C after the filtration step. The pellet was resuspended in 1 mL freezing media (10% DMSO, 90% FBS), then cryopreserved at −80°C.

Two panels were used to study both the myeloid and lymphoid lineages of hematopoietic immune cells. All antibodies were titrated prior to multiplexing and are described in Table 1. Unstained cells were used as a negative control in all experiments. Unstained controls and fluorescence-minus-one (FMO) controls were utilized to establish baseline gate settings for each respective antibody-fluorophore. For staining, cells were thawed and rinsed in cold PBS, centrifuged at 1,500 rpm for 5 min at 4°C, and supernatant decanted. Cells were counted using trypan blue and then seeded at 1 × 10^6^ live cells per well in 96-well V-bottom plates. Each sample was then incubated with Live/Dead aqua stain (Thermo Fisher Scientific) for 30 min in the dark at 4°C. FACS buffer (PBS +5% FBS +0.01% NaN_3_) was then added to each sample. Samples were centrifuged for 5 min at 1,500 rpm and supernatant decanted. Samples were then incubated in Fc blocking solution for 30 min in the dark at 4°C. Next, where appropriate, extracellular antibodies were added to the blocking solution. Samples were then incubated for 30 min in the dark at 4°C. FACS buffer was then added to each sample. Samples were centrifuged for 5 min at 1,500 rpm and decanted. After the samples were washed with FACS buffer, Cyto Fix/Perm solution was added (Biolegend). Samples were centrifuged for 5 min at 1,500 rpm and supernatant decanted. FACS buffer was then added to each sample. Samples were centrifuged for 5 min at 1,500 rpm and decanted. Perm/Wash solution was then added to each sample (Invitrogen), centrifuged for 5 min at 1,500 rpm and the supernatant discarded. Next, intracellular antibodies diluted in Perm/Wash solution were added and incubated for 30 min in the dark. Perm/Wash solution was then added to each sample. Samples were centrifuged for 5 min at 1,500 rpm and supernatant decanted. Samples were then washed with FACS buffer, centrifuged for 5 min at 1,500 rpm and supernatant decanted. Finally, pellets were resuspended in FACS buffer and were then analyzed using the BD LSR Fortessa (BD Biosciences, Lady Davis Institute Flow Cytometry Core). Flow cytometry data were then analyzed using FlowJo version 10.7. Forward scatter-H vs. forward scatter-A were used to gate out single cells only. The expanded gating strategy schemes for immune-cell types are shown in Supplementary Figures S1, S2.

**TABLE 1:** Antibodies for flow cytometry panels.

Antigen	Clone	Fluorochrome	Supplier, catalog #
T cell panel			
CD25	PC61	BV786	BD Biosciences, 564023
CD4	RM4-5	PerCP-e710	eBioscience, 46-0042-80
CD3	145-2C11	FITC	BD Biosciences, 553062
CD8	53–6.7	APC-e780	eBioscience, 47-0081-82
FoxP3	FJK-16s	APC	eBioscience, 17-5773-82
Myeloid and B cell panel			
CD45	30-F11	BUV395	BD Biosciences, 564279
CD11b	M1/70	e450	eBioscience, 48-0012-82
MHC II	M5/114.15.2	BV650	BD Biosciences, 563415
CD11c	HL3	BV786	BD Biosciences, 563735
Ly6G	1A8	FITC	BD Biosciences, 561105
F4/80	BM8	PE	eBioscience, 12-4801-80
Ly6C	AL-21	PE-CF594	BD Biosciences, 562728
CD19	MB19-1	APC	eBioscience, 17-0191-81

### 2.10 Statistical analyses

Statistics were performed using GraphPad Prism, version 8.00 (GraphPad Software, San Diego CA). The Shapiro-Wilk test was used to determine whether the data were normally distributed. A one-way or two-way ANOVA was used to indicate a significant difference as appropriate. Differences between groups were examined using multiple comparison test with Tukey correction for multiple comparisons. Data are shown as means ± SD. P <0.05 was considered statistically significant in all tests.

## 3 Results

### 3.1 Characterization of the AAV-PCSK9^DY^ murine atherosclerotic model post-JUUL exposure

Having established our murine exposure model of human vaping, we next combined this with an inducible atherosclerotic model. Standard in-bred strains of mice do not develop atherosclerosis, even on a high-fat diet (Getz and Reardon, 2012). Thus, in order to test whether JUUL exposure can enhance early pro-atherogenic changes, we utilized the AAV-PCSK9^DY^ inducible hyperlipidemic mouse model. Exposure to aerosolized PG/VG or JUUL did not further increase cholesterol levels (Figure 1B). We also measured the nicotine metabolite cotinine the day after the last exposure and surprisingly, found significant increases in cotinine levels in the JUUL-exposed mice compared to those exposed to air and PG/VG (Figure 1C) with levels ranging between 10 and 40 ng/mL. There was no difference in hepatic CYP2A5 protein expression among the exposure groups or with wild-type C57Bl6/6J mice (Figure 1D). Additionally, we measured the weight of the mice before (Day 0) and after the experiment (Day 150). After 4 weeks of aerosol exposure, there was a significant increase in the weight of mice exposed to room air (whereas mice exposed to PG/VG or JUUL failed to gain weight (Figure 1E).

### 3.2 Inhalation of JUUL aerosols in hyperlipidemic mice results in pro-atherogenic changes

**FIGURE 2:**
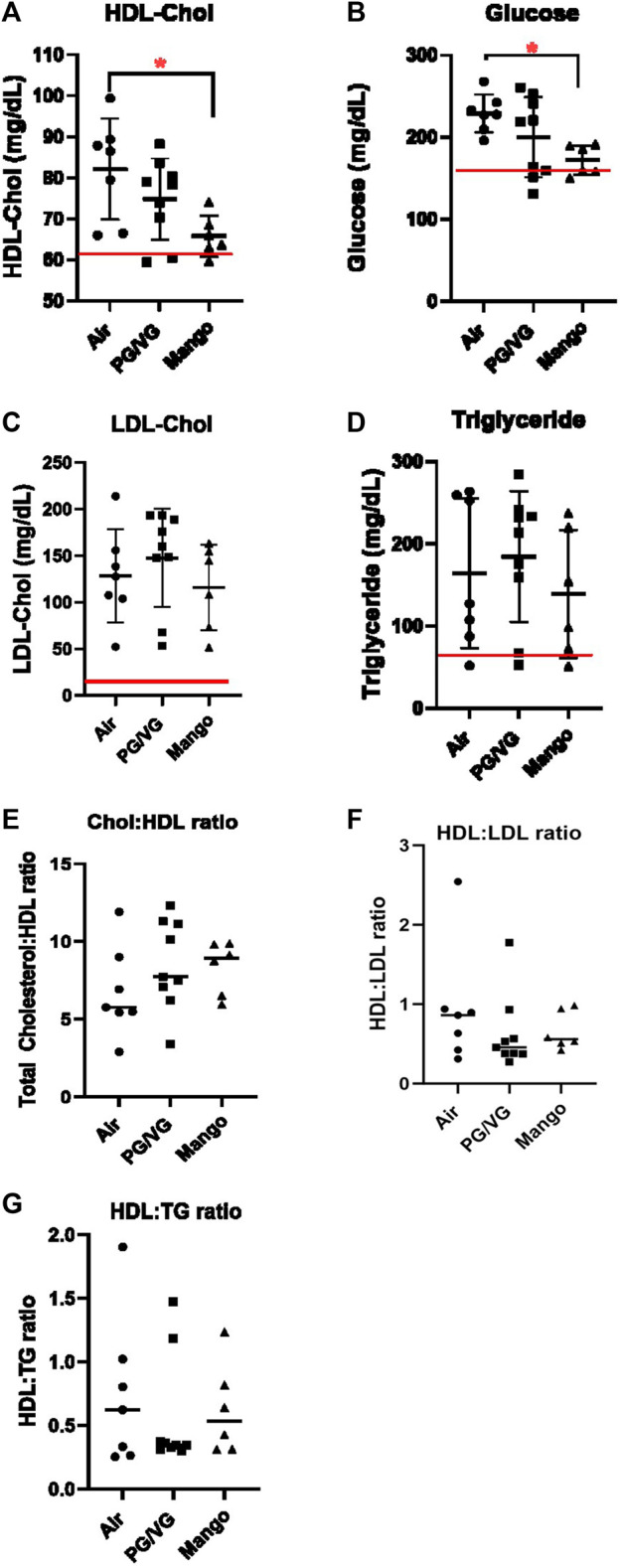
HDL-Chol **(A)** and glucose **(B)** levels are decreased in JUUL Mango-exposed AAV-PCSK9^DY^ mice compared to air control (* = p < 0.05). LDL-Chol **(C)** and triglycerides **(D)** were not altered. Red lines indicate average male C57BL/6 levels. HDL ratios are also shown in **(E–G)**. Results are expressed as the mean ± SD. *n* = 6–9.

In addition to cholesterol, we also measured circulating triglyceride, LDL, HDL, and glucose levels, as changes in these parameters are associated with increased vascular disease. AAV-PCSK9^DY^ infection alone altered lipid profiles from wild-type C57BL/6J mice [Figures 2A–D; red line indicates average male data (Otto et al., 2016; Jackson Laboratory, 2023)]. Significant decreases in both HDL and glucose levels were observed in JUUL-exposed mice compared to those that only received room air (Figures 2A, B); no changes were observed in triglycerides, LDL levels, or the ratios between total cholesterol:HDL, HDL:LDL, or HDL:triglycerides between exposure groups (Figures 2C–G).

**FIGURE 3:**
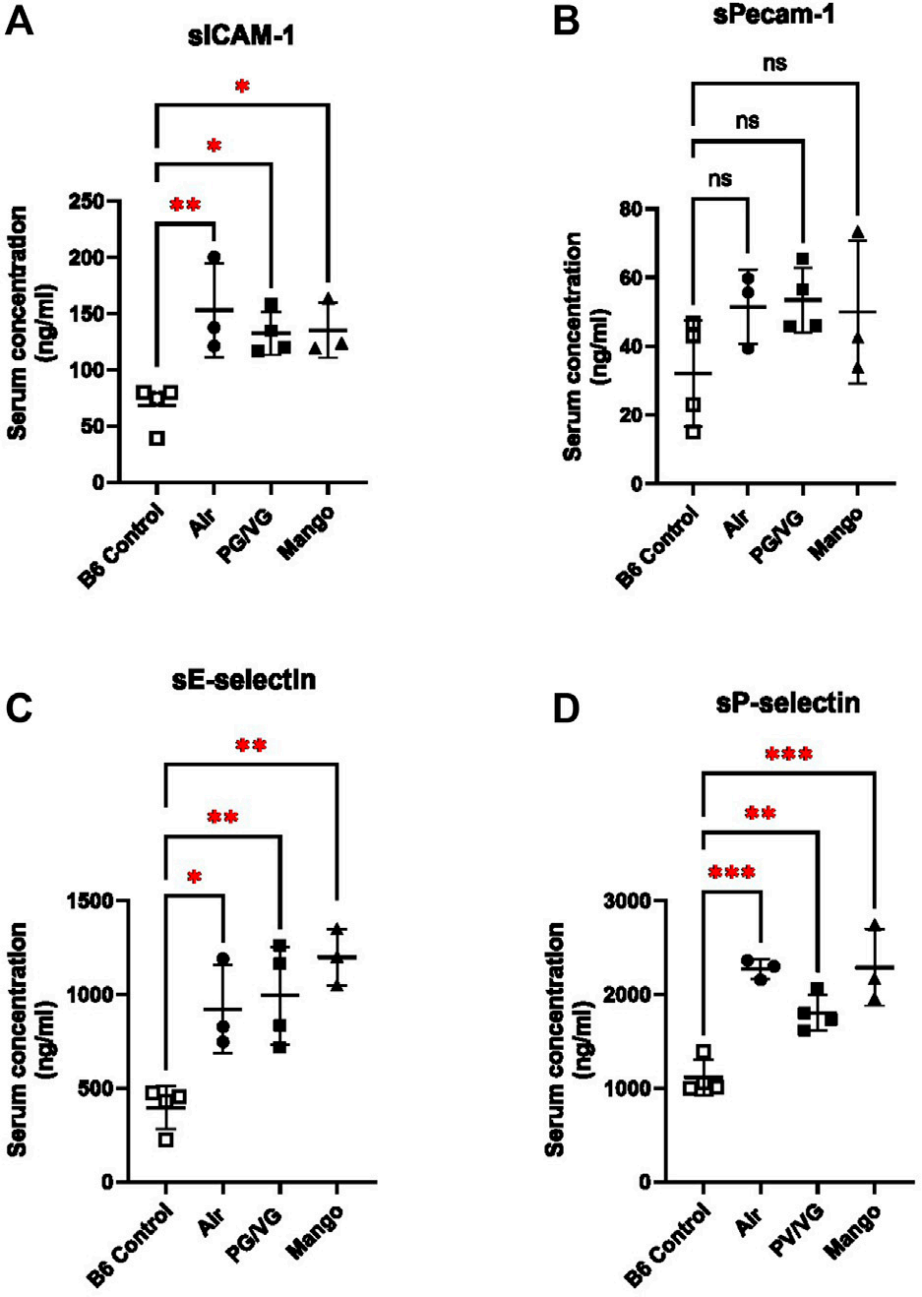
AAV-PCSK9^DY^ increases circulating soluble endothelial cell activation markers, which is not altered by JUUL aerosol exposure. Adhesion molecules **(A)** sICAM-1, **(B)** sPECAM-1, **(C)** sE-selectin, and **(D)** sP-selectin were assessed in the serum of control (open squares) and hyperlipidemic mice via multi-plex ELISA. AAV-PCSK9^DY^ mice were exposed to air (black circles), PG/VG (black squares), or Mango-flavored JUUL (black triangles). Soluble adhesion molecule levels for uninfected, unexposed C57BL/6J mice are shown for comparison. Results are expressed as the mean ± SD. *n* = 3–4.

**FIGURE 4:**
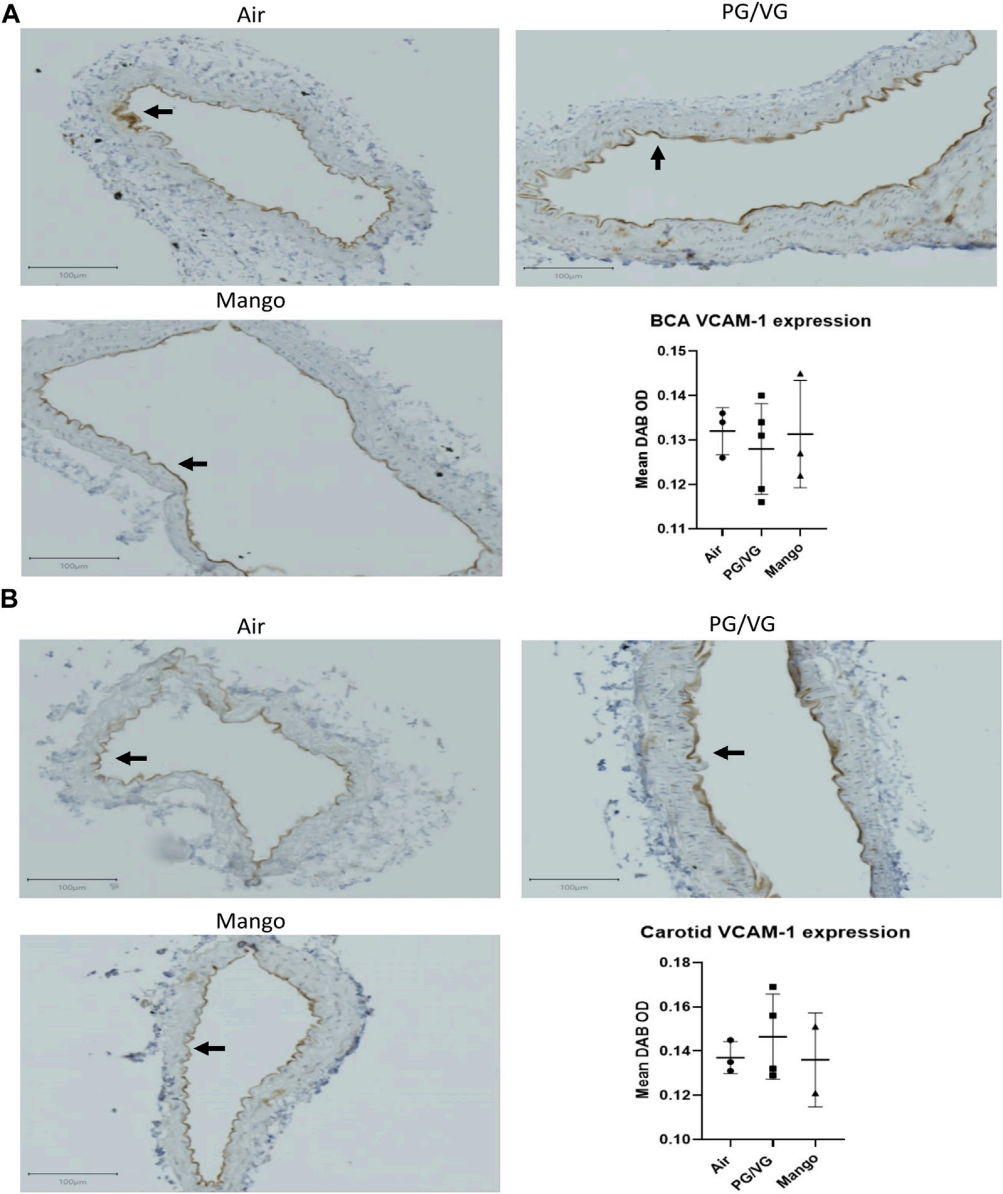
JUUL aerosol exposure has no effect on VCAM-1 expression on the endothelial cells in either the BCA **(A)** or carotid **(B)** as assessed by IHC staining and quantified by measuring Mean DAB OD within the endothelial cell layer. Results are expressed as the mean ± SD. *n* = 2–4.

Next, we evaluated early markers of endothelial dysfunction that contribute to atherosclerosis and are biomarkers of CVD. First, we assessed soluble markers of endothelial cell activation (ICAM-1, PECAM-1, E-selectin, P-selectin, MMP-9, and PAI-1) in the serum, comparing AAV-PCSK9^DY^-infected C57BL/6J mice exposed to air, PG/VG and JUUL with unexposed, uninfected wild-type mice. Levels of MMP-9 and PAI-1 were below the limit of detection in all mice (data not shown). The levels of all detectable markers were higher in the hyperlipidemic mice compared to wild type mice with three being statistically significant: ICAM-1, P-selectin, and E-selectin (Figure 3). However, no changes were observed amongst exposure groups as compared to air (Figure 3). In addition, we assessed VCAM-1 expression on the endothelium lining the BCA and carotid arteries of the AAV-PCSK9^DY^-infected mice using IHC. No significant differences were observed in either the BCA (Figure 4A) or carotid arteries (Figure 4B).

**FIGURE 5:**
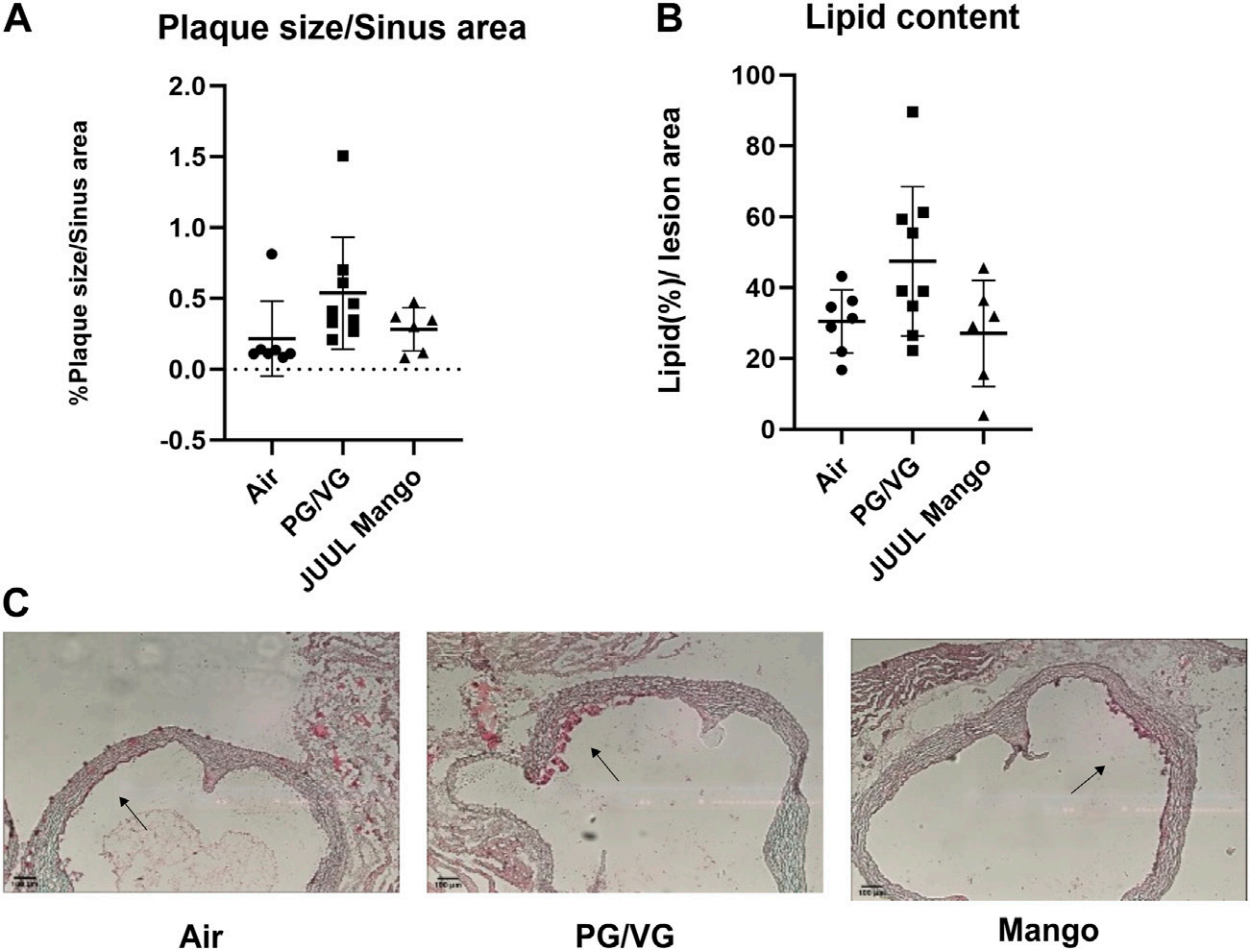
No significant changes were observed in plaque size within the aortic sinus. **(A)** Plaque size (plaque per sinus area) and **(B)** lipid content (staining per plaque area) were quantified in the aortic sinus after oil red O staining. Representative images from each group are shown in **(C)**. Black arrows point toward small fatty streaks/plaques. Results are expressed as the mean ± SD. *n* = 6–9.

In murine models, atherosclerotic plaque development requires around 9–15 weeks depending on diet (Nakashima et al., 1994); thus we did not anticipate large plaques after only 4–5 weeks. Indeed, 4 weeks after the injection of the AAV-PCSK9^DY^ the aortic sinus plaques in all mice were small but detectable. Moreover, there was no significant difference between the exposure groups, although there was a trend of increased plaque size in the PG/VG-exposed mice compared to the control group or the JUUL-exposed group (*p* = 0.1; Figure 5A). Moreover, the percentage of lipid content within the plaque was not different between groups, although the same trend of increased lipid content in the PG/VG-exposed group compared to the control group was observed (Figures 5B, C).

### 3.3 Inhalation of JUUL aerosols increases indices of systemic inflammation

**FIGURE 6:**
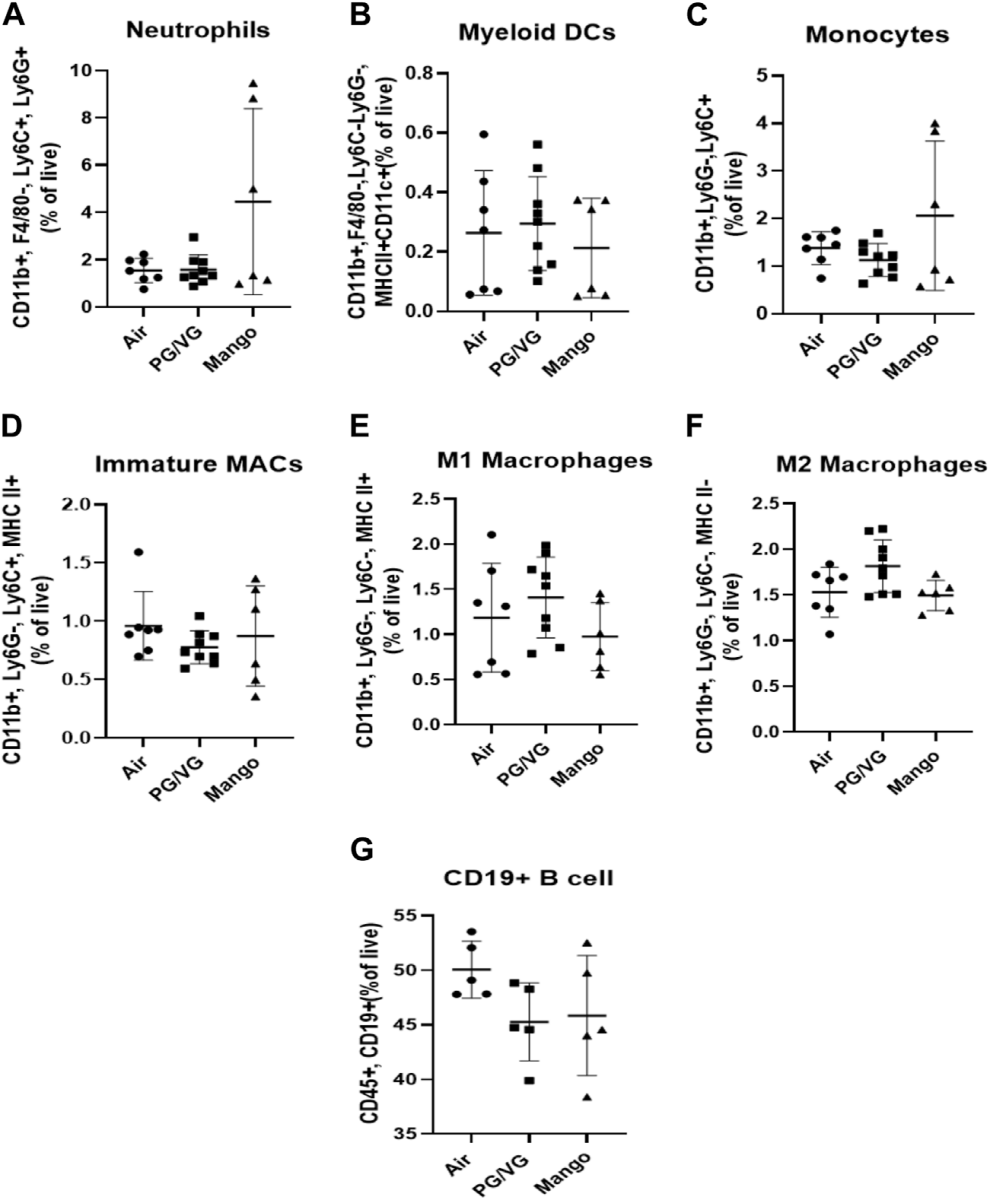
Effect of JUUL aerosol exposure on immune cell distribution of myeloid cells and B cells in the spleen. Using immunophenotyping, the following cell populations were identified: **(A)** neutrophils, **(B)** myeloid DCs, **(C)** monocytes, **(D–F)** immature, M1, and M2 macrophages, and **(G)** B cells. Results are expressed as the mean ± SD; individual data points represent individual mice. *n* = 6–9.

**FIGURE 7:**
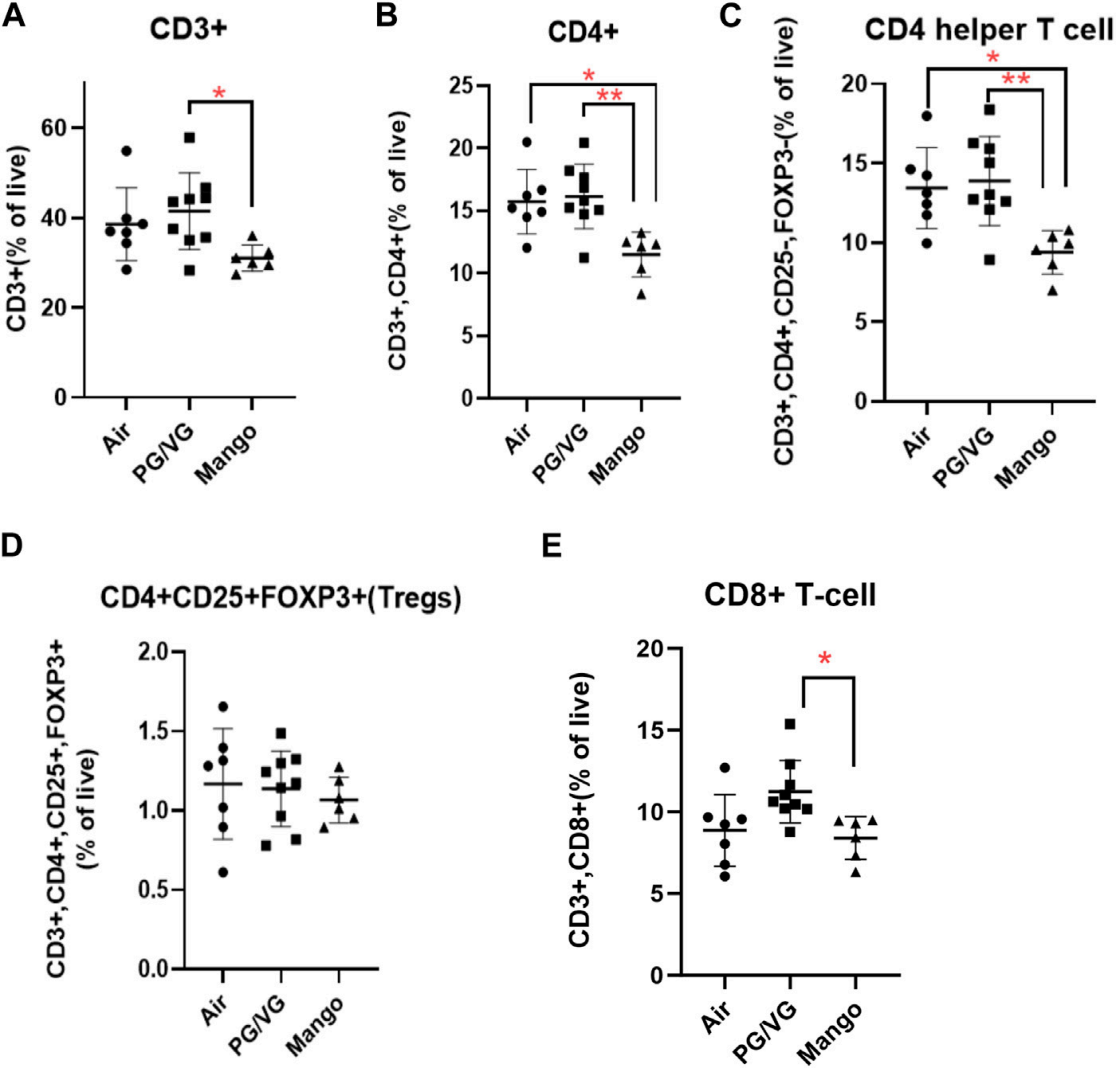
Effect of JUUL aerosol exposure on T cell distribution in the spleen. Using immunophenotyping, splenic T cell populations were analyzed as follows: **(A)** CD3^+^ total T-cells, **(B)** CD4^+^ T cells, **(C)** CD4 helper T cells, **(D)** CD25^+^ FoxP3+Tregs, and **(E)** CD8^+^ T cells. Results are expressed as the mean ± SD; individual data points represent individual mice. (* = p ˂ 0.05, ** = p ˂ 0.005) *n* = 6–9.

Given that atherosclerosis is caused by systemic inflammation (Wolf and Ley, 2019), we sought to assess whether JUUL exposure would alter immune cell populations by immunophenotyping splenic cells to assess myeloid and lymphoid lineages. No significant changes were observed in myeloid cell subsets or B cell populations within the spleen (Figure 6). However, the percentage of total CD3^+^ T cells was decreased by exposure to JUUL compared to PG/VG (Figure 7A). CD4^+^ and CD4^+^ helper T cells (Th cells) were also significantly decreased following JUUL exposure compared to both room air and PG/VG (Figures 7B, C). The percentage of CD4^+^ regulatory T cells (Tregs) was not altered by any exposure (Figure 7D). Finally, the percentage of CD8^+^ cytotoxic T cells in the PG/VG group was significantly increased compared to mice exposed to JUUL (Figure 7E). Together, our data showed that exposure to PG/VG and JUUL aerosols causes changes significant changes in splenic T cell subsets.

## 4 Discussion

In this study, we utilized an AAV-PCSK9^DY^ inducible model of atherosclerosis in conjunction with our established vaping protocol (Been et al., 2022) as a means to study vascular pathology. The use of e-cigarettes has been associated with negative cardiovascular outcomes (Lee et al., 2018) and immunological responses (Hwang et al., 2016; Corriden et al., 2020), but the results thus far from atherosclerotic mouse models have been contradictory (Espinoza-Derout et al., 2019; Szostak et al., 2020). Standard in-bred strains of mice are highly resistant to the development of atherosclerosis (Meir and Leitersdorf, 2004; Getz and Reardon, 2012); therefore, genetic modification of their lipid metabolism is required (i.e., through deletion of ApoE or LDL-R genes). Our findings show that the AAV-PCSK9^DY^ (hyperlipidemic) model is highly effective for studying the pro-atherogenic effects of vaping. Importantly, we further showed using this model that there are modest changes in early proatherogenic indicators at this relatively short length of time (4 weeks), suggesting that assessment of atherosclerosis at longer exposure levels may be important for the assessment of CVD in vaping populations. Importantly, cotinine levels observed herein are within the range observed in e-cigarette users (Flouris et al., 2013; Behar et al., 2015; Marsot and Simon, 2016).

A single injection of AAV-PCSK9^DY^ is sufficient to cause downregulation of LDL-R, inducing hyperlipidemia and atherosclerosis in mice, where plaque formation is similar to humans (Roche-Molina et al., 2015; Goettsch et al., 2016). This model has the advantage over the more established transgenic atherosclerosis mouse models (i.e., ApoE^−/−^ and LDL-R^−/−^ mice) in that it allows for control over the time of onset of hyperlipidemia in the lifespan of the animal. Establishing this inducible-atherosclerosis model was therefore an important first step toward testing real-world exposure scenarios. For example, we can now model whether JUUL use in adolescence predisposes individuals to cardiovascular risk later in life, when lifestyle choices, such as a high fat diet or smoking, combine to raise the risk. One could initiate vaping in young mice (before injection with AAV-PCSK9^DY^ to induce hyperlipidemia) and with and without a high fat diet and assess whether there are any variations in cardiopulmonary outcomes. This would mimic the current age demographic of vapers to better understand if/how youth vaping predisposes to CVD risk later in life when combined with poor lifestyle choices (e.g., high-fact diet). This model can also allow us and others the ability to ascertain, for example, whether switching from cigarette smoking to JUUL (in conjunction with the AAV-PCSK9^DY^) reduces cardiovascular risk by attenuating pulmonary and systemic inflammation. This question is highly relevant to current smokers who are using e-cigarettes as a smoking-cessation tool, where up to 80% of those that quit smoking were still vaping 1 year later (Hajek et al., 2019).

One early indicator of CVD is dyslipidemia (Miller, 2009), defined as an imbalance of lipids such as cholesterol, LDL-C, triglycerides and HDL. Low HDL in particular is a component of metabolic syndrome and is common in individuals with coronary artery disease. Cigarette smoking is also associated with decreasing HDL levels. Herein, we show that HDL levels were significantly decreased after a 4-week exposure to JUUL mango in this hyperlipidemic mouse model. HDL plays an important role in the clearance of cholesterol from tissues (Linton et al., 2000) and maintains plaque stability by inhibiting degradation of the fibrous cap extracellular matrix through its anti-elastase activity (Ortiz-Muñoz et al., 2009). Thus, decreased HDL levels could indicate that JUUL exposure results in insufficient clearance of cholesterol from tissues, although at 1 month we do not see an accumulation of lipid within the artery. In contrast, JUUL exposure decreased glucose levels. Diabetes is clearly linked to an increased risk of CVD, however, an association between glucose levels and CVD is less clear. However, in non-diabetic patients, increased glucose levels corresponded to increased CVD risk (Riise et al., 2021). Our data that indicate that JUUL may not impact CVD through increased glucose levels.

One interesting observation from this study is that mice exposed to either PG/VG or JUUL failed to gain weight during the course of the exposure. Our findings are consistent with a previous report on the effect of short (2 weeks) e-cigarette exposure on cardiac function and body weight in C57BL/6J mice, where they found that e-cigarette exposure significantly inhibited bodyweight gain (Shi et al., 2019). Moreover, our findings are similar to what was observed after 4 weeks in ApoE^−/−^ mice exposed to e-cigarette aerosols and fed high fat-diet for 12 weeks, although the decrease in weight was only observed in the nicotine (2.4%) group and was transient (Espinoza-Derout et al., 2019). Tobacco smoking is associated with lower body weight and nicotine alters energy balance (Seoane-Collazo et al., 2021), but it is unclear if e-cigarettes will have the same effect on body weight (Hod et al., 2022). Lack of weight gain may be due to decreased food consumption (Shao et al., 2019) and/or the presence of nicotine, both which could be measured carefully in future experiments.

Another important indicator of atherogenesis is an increase in systemic inflammation (Wolf and Ley, 2019). Our immunoprofiling data showed that JUUL exposure altered specific splenic immune cell populations, specifically a decrease in CD4^+^ Th cells. CD4^+^ Th cells can be either pro- or anti-atherogenic, depending upon their polarization towards Th1 or Th2 cells, respectively (Wolf and Ley, 2019). Future investigation should focus on further characterization of T cell subtypes as well as T cell function and activation, as both are negatively regulated by HDL (Grao-Cruces et al., 2022). HDL levels were decreased following JUUL exposure, potentially increasing T cell activation. Unlike T cells, myeloid lineage cells, such as neutrophils, monocytes, and macrophages, as well as B cells, did not change after vaping, but modulation of HDL levels could alter their function. Thus, e-cigarette exposure leads to HDL levels in this AAV-PCSK9^DY^ model, which may lead to important pro-atherogenic immune modulation over time. An important component of future studies will be assessment of the immune components of the plaque, in addition to the systemic immune response.

Although many of the substances in e-liquids are “Generally Recognized as Safe” for oral intake, including PG/VG and flavour chemicals, there is little data on their safety when aerosolized and inhaled (Kaur et al., 2018). PG/VG is one of the most common solvents used in e-cigarette and by itself can be toxic and reduce cell viability *in vitro* (Sassano et al., 2018). Moreover, aerosolization of these solvents is associated with the generation of harmful chemicals, including diacetyl, formaldehyde, and methylglyoxal (Azimi et al., 2021; Tehrani et al., 2021). PG/VG aerosols may also include other components including metals derived from coil breakdown (Aherrera et al., 2017; Olmedo et al., 2018). Our data revealed some alterations are caused by PG/VG in the absence of other chemicals (including nicotine); this included the suppression of weight gain as well as a trend toward increased plaque size and CD8^+^ T cells in the spleen, both of which were not observed in mango-flavored JUUL exposure. These findings suggest that PG/VG is not inert and has the potential to affect important physiological responses that may predispose to disease risk. Importantly, it emphasizes the need to study the “real-world” devices/e-liquids.

There are several limitations to our study. We intended the study as a proof-of-principle to determine whether the AAV-PCSK9^DY^ model would be amenable to study e-cigarette exposure before embarking on the significantly longer study required to assess atherosclerotic plaque. Thus, this study does not provide information on dose response, nor does it provide information on other devices or flavors. We utilized mango-flavored JUUL containing 59 mg/mL nicotine, because at the initiation of the experiment, it was the most common product in our market. However, Québec, along with many other districts, has now restricted the amount of nicotine and flavored-products (other than tobacco) (Laframboise, 2023). Longer term studies should be performed using the most current products, that would compare doses (i.e., puff frequency) and longer time points, but also consider the contribution of nicotine versus other additives to the e-liquid.

Together, our results demonstrate that early signs of atherosclerosis are induced in the AAV-PCSK9^DY^ inducible hyperlipidemic model following exposure to e-cigarettes. Generation of this model will allow us to interrogate further the development of atherosclerosis over longer exposure times and in addition, will facilitate separation of the vaping and hyperlipidemic exposures to better model human exposures.

## Data Availability

The original contributions presented in the study are included in the article/Supplementary Material, further inquiries can be directed to the corresponding author.

## Abbreviations

ApoE, apolipoprotein E; AAV-PCSK9, adeno-associated virus- protein convertase subtilisin/kexin type 9; BCA, brachiocephalic arch; CVD, cardiovascular disease; PG, propylene glycol; VG, vegetable glycerin; LDL, low-density lipoprotein; HDL, levels and decreased high density lipoprotein; matrix metalloproteinase-9, MMP-9; ROS, reactive oxygen species; VCAM-1, vascular cell adhesion molecule 1.

## References

[B1] Aherrera A. Olmedo P. Grau-Perez M. Tanda S. Goessler W. Jarmul S. (2017). The association of e-cigarette use with exposure to nickel and chromium: a preliminary study of non-invasive biomarkers. Environ. Res. 159, 313–320. 10.1016/j.envres.2017.08.014 28837903

[B2] Ahmad S. Wang T. Schwartz R. Bondy S. J. (2022). Predictors of pod-type e-cigarette device use among Canadian youth and young adults. Health Promot Chronic Dis. Prev. Can. 42, 12–20. 10.24095/hpcdp.42.1.03 35044140 PMC9067011

[B3] Azimi P. Keshavarz Z. Lahaie Luna M. Cedeno Laurent J. G. Vallarino J. Christiani D. C. (2021). An unrecognized hazard in E-cigarette vapor: preliminary quantification of methylglyoxal formation from propylene glycol in E-cigarettes. Int. J. Environ. Res. Public Health 18, 385. 10.3390/ijerph18020385 33419122 PMC7825490

[B4] Been T. Alakhtar B. Traboulsi H. Tsering T. Bartolomucci A. Heimbach N. (2023). Chronic low-level JUUL aerosol exposure causes pulmonary immunologic, transcriptomic, and proteomic changes. FASEB J. 37, e22732. 10.1096/fj.202201392R 36694994

[B5] Been T. Traboulsi H. Paoli S. Alakhtar B. Mann K. K. Eidelman D. H. (2022). Differential impact of JUUL flavors on pulmonary immune modulation and oxidative stress responses in male and female mice. Archives Toxicol. 96, 1783–1798. 10.1007/s00204-022-03269-3 35254488

[B6] Behar R. Z. Hua M. Talbot P. (2015). Puffing topography and nicotine intake of electronic cigarette users. PLoS One 10, e0117222. 10.1371/journal.pone.0117222 25664463 PMC4321841

[B7] Cahn Z. Siegel M. (2011). Electronic cigarettes as a harm reduction strategy for tobacco control: a step forward or a repeat of past mistakes? J. Public Health Policy 32, 16–31. 10.1057/jphp.2010.41 21150942

[B8] Caponnetto P. Campagna D. Papale G. Russo C. Polosa R. (2012). The emerging phenomenon of electronic cigarettes. Expert Rev. Respir. Med. 6, 63–74. 10.1586/ers.11.92 22283580

[B9] Civiletto C. W. Hutchison J. (2019). Electronic vaping delivery of cannabis and nicotine. 31424744

[B10] Civiletto C. W. Hutchison J. (2022). Electronic vaping delivery of cannabis and nicotine. *StatPearls* . Treasure Island (FL): StatPearls Publishing Copyright © 2022. StatPearls Publishing LLC. 31424744

[B11] Corriden R. Moshensky A. Bojanowski C. M. Meier A. Chien J. Nelson R. K. (2020). E-cigarette use increases susceptibility to bacterial infection by impairment of human neutrophil chemotaxis, phagocytosis, and NET formation. Am. J. Physiol. Cell Physiol. 318, C205-C214–C214. 10.1152/ajpcell.00045.2019 31664858 PMC6985828

[B12] Espinoza-Derout J. Hasan K. M. Shao X. M. Jordan M. C. Sims C. Lee D. L. (2019). Chronic intermittent electronic cigarette exposure induces cardiac dysfunction and atherosclerosis in apolipoprotein-E knockout mice. Am. J. Physiology-Heart Circulatory Physiology 317, H445-H459–H459. 10.1152/ajpheart.00738.2018 PMC6732484 31172811

[B13] Fadus M. C. Smith T. T. Squeglia L. M. (2019). The rise of e-cigarettes, pod mod devices, and JUUL among youth: factors influencing use, health implications, and downstream effects. Drug Alcohol Depend. 201, 85–93. 10.1016/j.drugalcdep.2019.04.011 31200279 PMC7183384

[B14] Falk E. (2006). Pathogenesis of atherosclerosis. J. Am. Coll. Cardiol. 47, C7–C12. 10.1016/j.jacc.2005.09.068 16631513

[B15] Flouris A. D. Chorti M. S. Poulianiti K. P. Jamurtas A. Z. Kostikas K. Tzatzarakis M. N. (2013). Acute impact of active and passive electronic cigarette smoking on serum cotinine and lung function. Inhal. Toxicol. 25, 91–101. 10.3109/08958378.2012.758197 23363041

[B16] Getz G. S. Reardon C. A. (2012). Animal models of atherosclerosis. Arterioscler. Thromb. Vasc. Biol. 32, 1104–1115. 10.1161/ATVBAHA.111.237693 22383700 PMC3331926

[B17] Goettsch C. Hutcheson J. D. Hagita S. Rogers M. A. Creager M. D. Pham T. (2016). A single injection of gain-of-function mutant PCSK9 adeno-associated virus vector induces cardiovascular calcification in mice with no genetic modification. Atherosclerosis 251, 109–118. 10.1016/j.atherosclerosis.2016.06.011 27318830 PMC4983246

[B18] Goniewicz M. L. Boykan R. Messina C. R. Eliscu A. Tolentino J. (2019). High exposure to nicotine among adolescents who use Juul and other vape pod systems (‘pods’). Tob. control 28, 676–677. 10.1136/tobaccocontrol-2018-054565 30194085 PMC6453732

[B19] Grao-Cruces E. Lopez-Enriquez S. Martin M. E. Montserrat-De La Paz S. (2022). High-density lipoproteins and immune response: a review. Int. J. Biol. Macromol. 195, 117–123. 10.1016/j.ijbiomac.2021.12.009 34896462

[B20] Hajek P. Phillips-Waller A. Przulj D. Pesola F. Myers Smith K. Bisal N. (2019). A randomized trial of E-cigarettes versus nicotine-replacement therapy. N. Engl. J. Med. 380, 629–637. 10.1056/NEJMoa1808779 30699054

[B21] Hammond D. Reid J. L. Rynard V. L. Fong G. T. Cummings K. M. Mcneill A. (2019). Prevalence of vaping and smoking among adolescents in Canada, England, and the United States: repeat national cross sectional surveys. BMJ 365, l2219. 10.1136/bmj.l2219 31221636 PMC6582265

[B22] Hod R. Mohd Nor N. H. Maniam S. (2022). Systematic review on e-cigarette and its effects on weight gain and adipocytes. PLoS One 17, e0270818. 10.1371/journal.pone.0270818 35788209 PMC9255744

[B23] Hwang J. H. Lyes M. Sladewski K. Enany S. Mceachern E. Mathew D. P. (2016). Electronic cigarette inhalation alters innate immunity and airway cytokines while increasing the virulence of colonizing bacteria. J. Mol. Med. Berl. 94, 667–679. 10.1007/s00109-016-1378-3 26804311

[B24] Hwang S. J. Ballantyne C. M. Sharrett A. R. Smith L. C. Davis C. E. Gotto A. M. JR. (1997). Circulating adhesion molecules VCAM-1, ICAM-1, and E-selectin in carotid atherosclerosis and incident coronary heart disease cases: the Atherosclerosis Risk in Communities (ARIC) study. Circulation 96, 4219–4225. 10.1161/01.cir.96.12.4219 9416885

[B25] Jackler R. K. Ramamurthi D. (2019). Nicotine arms race: JUUL and the high-nicotine product market. Tob. Control 28, 623–628. 10.1136/tobaccocontrol-2018-054796 30733312

[B28] Jackson Laboratory (2023). Mouse phenome database: measure involving lipid profile. Available: https://phenome.jax.org/procedures/lipid%20profile (Accessed).

[B26] Kaur G. Muthumalage T. Rahman I. (2018). Mechanisms of toxicity and biomarkers of flavoring and flavor enhancing chemicals in emerging tobacco and non-tobacco products. Toxicol. Lett. 288, 143–155. 10.1016/j.toxlet.2018.02.025 29481849 PMC6549714

[B27] King B. A. Gammon D. G. Marynak K. L. Rogers T. (2018). Electronic cigarette sales in the United States, 2013-2017. JAMA 320, 1379–1380. 10.1001/jama.2018.10488 30285167 PMC6233837

[B29] Laframboise K. (2023). Quebec’s ban on sale of flavoured vapes is now in effect. Here is what you need to know. Available: https://globalnews.ca/news/10060555/quebec-flavour-vapes-ban-october-31-2023/(Accessed).

[B30] Leavens E. L. S. Stevens E. M. Brett E. I. Hebert E. T. Villanti A. C. Pearson J. L. (2019). JUUL electronic cigarette use patterns, other tobacco product use, and reasons for use among ever users: results from a convenience sample. Addict. Behav. 95, 178–183. 10.1016/j.addbeh.2019.02.011 30933713

[B31] Lee H. W. Park S. H. Weng M. W. Wang H. T. Huang W. C. Lepor H. (2018). E-cigarette smoke damages DNA and reduces repair activity in mouse lung, heart, and bladder as well as in human lung and bladder cells. Proc. Natl. Acad. Sci. U. S. A. 115, E1560-E1569–e1569. 10.1073/pnas.1718185115 29378943 PMC5816191

[B32] Lemaire M. Lemarie C. A. Molina M. F. Schiffrin E. L. Lehoux S. Mann K. K. (2011). Exposure to moderate arsenic concentrations increases atherosclerosis in ApoE-/- mouse model. Toxicol. Sci. 122, 211–221. 10.1093/toxsci/kfr097 21512104 PMC3143470

[B33] Linton M. F. Yancey P. G. Davies S. S. Jerome W. G. Linton E. F. Song W. L. (2000). “The role of lipids and lipoproteins in atherosclerosis,” in Endotext. Editors FEINGOLD K. R. ANAWALT B. BOYCE A. CHROUSOS G. DE HERDER W. W. DHATARIYA K. (South Dartmouth (MA): MDText.com, Inc). Copyright © 2000-2022, MDText.com, Inc.

[B34] Marsot A. Simon N. (2016). Nicotine and cotinine levels with electronic cigarette: a review. Int. J. Toxicol. 35, 179–185. 10.1177/1091581815618935 26681385

[B35] Maxwell K. N. Breslow J. L. (2004). Adenoviral-mediated expression of Pcsk9 in mice results in a low-density lipoprotein receptor knockout phenotype. Proc. Natl. Acad. Sci. U. S. A. 101, 7100–7105. 10.1073/pnas.0402133101 15118091 PMC406472

[B36] Meir K. S. Leitersdorf E. (2004). Atherosclerosis in the apolipoprotein-E-deficient mouse: a decade of progress. Arterioscler. Thromb. Vasc. Biol. 24, 1006–1014. 10.1161/01.ATV.0000128849.12617.f4 15087308

[B37] Miller M. (2009). Dyslipidemia and cardiovascular risk: the importance of early prevention. QJM Int. J. Med. 102, 657–667. 10.1093/qjmed/hcp065 PMC2729130 19498039

[B38] Nakashima Y. Plump A. S. Raines E. W. Breslow J. L. Ross R. (1994). ApoE-deficient mice develop lesions of all phases of atherosclerosis throughout the arterial tree. Arterioscler. Thromb. 14, 133–140. 10.1161/01.atv.14.1.133 8274468

[B39] Olmedo P. Goessler W. Tanda S. Grau-Perez M. Jarmul S. Aherrera A. (2018). Metal concentrations in e-cigarette liquid and aerosol samples: the contribution of metallic coils. Environ. Health Perspect. 126, 027010. 10.1289/EHP2175 29467105 PMC6066345

[B40] Ortiz-Muñoz G. Houard X. Martín-Ventura J. L. Ishida B. Y. Loyau S. Rossignol P. (2009). HDL antielastase activity prevents smooth muscle cell anoikis, a potential new antiatherogenic property. Faseb J. 23, 3129–3139. 10.1096/fj.08-127928 19417089 PMC2735359

[B41] Otto G. P. Rathkolb B. Oestereicher M. A. Lengger C. J. Moerth C. Micklich K. (2016). Clinical chemistry reference intervals for C57bl/6J, C57bl/6N, and C3HeB/FeJ mice (*Mus musculus*). J. Am. Assoc. Lab. Anim. Sci. 55, 375–386. 27423143 PMC4943607

[B42] Pankow J. F. Kim K. Mcwhirter K. J. Luo W. Escobedo J. O. Strongin R. M. (2017). Benzene formation in electronic cigarettes. PloS one 12, e0173055. 10.1371/journal.pone.0173055 28273096 PMC5342216

[B43] Rao P. Liu J. Springer M. L. (2020). JUUL and combusted cigarettes comparably impair endothelial function. Tob. Regul. Sci. 6, 30–37. 10.18001/TRS.6.1.4 31930162 PMC6953758

[B44] Riise H. K. R. Igland J. Sulo G. Graue M. Haltbakk J. Tell G. S. (2021). Casual blood glucose and subsequent cardiovascular disease and all-cause mortality among 159 731 participants in Cohort of Norway (CONOR). BMJ Open Diabetes Res. Care 9, e001928. 10.1136/bmjdrc-2020-001928 PMC7907851 33622686

[B45] Roche-Molina M. Sanz-Rosa D. Cruz F. M. Garcia-Prieto J. Lopez S. Abia R. (2015). Induction of sustained hypercholesterolemia by single adeno-associated virus-mediated gene transfer of mutant hPCSK9. Arterioscler. Thromb. Vasc. Biol. 35, 50–59. 10.1161/ATVBAHA.114.303617 25341796

[B46] Sassano M. F. Davis E. S. Keating J. E. Zorn B. T. Kochar T. K. Wolfgang M. C. (2018). Evaluation of e-liquid toxicity using an open-source high-throughput screening assay. PLoS Biol. 16, e2003904. 10.1371/journal.pbio.2003904 29584716 PMC5870948

[B47] Seoane-Collazo P. Dieguez C. Nogueiras R. Rahmouni K. Fernandez-Real J. M. Lopez M. (2021). Nicotine' actions on energy balance: friend or foe? Pharmacol. Ther. 219, 107693. 10.1016/j.pharmthera.2020.107693 32987056

[B48] Shao X. M. Lopez B. Nathan D. Wilson J. Bankole E. Tumoyan H. (2019). A mouse model for chronic intermittent electronic cigarette exposure exhibits nicotine pharmacokinetics resembling human vapers. J. Neurosci. Methods 326, 108376. 10.1016/j.jneumeth.2019.108376 31361999 PMC6717674

[B49] Shi H. Fan X. Horton A. Haller S. T. Kennedy D. J. Schiefer I. T. (2019). The effect of electronic-cigarette vaping on cardiac function and angiogenesis in mice. Sci. Rep. 9, 4085–4089. 10.1038/s41598-019-40847-5 30858470 PMC6411855

[B50] Szostak J. Wong E. T. Titz B. Lee T. Wong S. K. Low T. (2020). A 6-month systems toxicology inhalation study in ApoE−/− mice demonstrates reduced cardiovascular effects of E-vapor aerosols compared with cigarette smoke. Am. J. Physiology-Heart Circulatory Physiology 318, H604-H631–H631. 10.1152/ajpheart.00613.2019 31975625

[B51] Tehrani M. W. Newmeyer M. N. Rule A. M. Prasse C. (2021). Characterizing the chemical landscape in commercial E-cigarette liquids and aerosols by liquid chromatography–high-resolution mass spectrometry. Chem. Res. Toxicol. 34, 2216–2226. 10.1021/acs.chemrestox.1c00253 34610237 PMC11317110

[B52] Traboulsi H. Cherian M. Abou Rjeili M. Preteroti M. Bourbeau J. Smith B. M. (2020). Inhalation toxicology of vaping products and implications for pulmonary health. Int. J. Mol. Sci. 21, 3495. 10.3390/ijms21103495 32429092 PMC7278963

[B53] Vindhyal M. R. Ndunda P. Munguti C. Vindhyal S. Okut H. (2019). Impact on cardiovascular outcomes among e-cigarette users: a review from National Health Interview Surveys. J. Am. Coll. Cardiol. 73, 11. 10.1016/s0735-1097(19)33773-8

[B54] Vogel E. A. Prochaska J. J. Ramo D. E. Andres J. Rubinstein M. L. (2019). Adolescents' E-cigarette use: increases in frequency, dependence, and nicotine exposure over 12 months. J. Adolesc. Health 64, 770–775. 10.1016/j.jadohealth.2019.02.019 31122507 PMC6538303

[B55] Vozenilek A. E. Blackburn C. M. R. Schilke R. M. Chandran S. Castore R. Klein R. L. (2018). AAV8-mediated overexpression of mPCSK9 in liver differs between male and female mice. Atherosclerosis 278, 66–72. 10.1016/j.atherosclerosis.2018.09.005 30253291 PMC6263847

[B56] Willett J. G. Bennett M. Hair E. C. Xiao H. Greenberg M. S. Harvey E. (2019). Recognition, use and perceptions of JUUL among youth and young adults. Tob. Control 28, 115–116. 10.1136/tobaccocontrol-2018-054273 29669749

[B57] Wolf D. Ley K. (2019). Immunity and inflammation in atherosclerosis. Circ. Res. 124, 315–327. 10.1161/CIRCRESAHA.118.313591 30653442 PMC6342482

[B58] Zhao D. Aravindakshan A. Hilpert M. Olmedo P. Rule A. M. Navas-Acien A. (2020). Metal/metalloid levels in electronic cigarette liquids, aerosols, and human biosamples: a systematic review. Environ. Health Perspect. 128, 36001. 10.1289/EHP5686 32186411 PMC7137911

