# Supplementary material for: E-cigarette exposure causes early pro-atherogenic changes in an inducible murine model of atherosclerosis
Source: Front Toxicol. 2023 Dec 18;5:1244596. doi: 10.3389/ftox.2023.1244596 (PMC10757938; doi:10.3389/ftox.2023.1244596)

Supplementary figure 1. T cell panel Gating Strategy

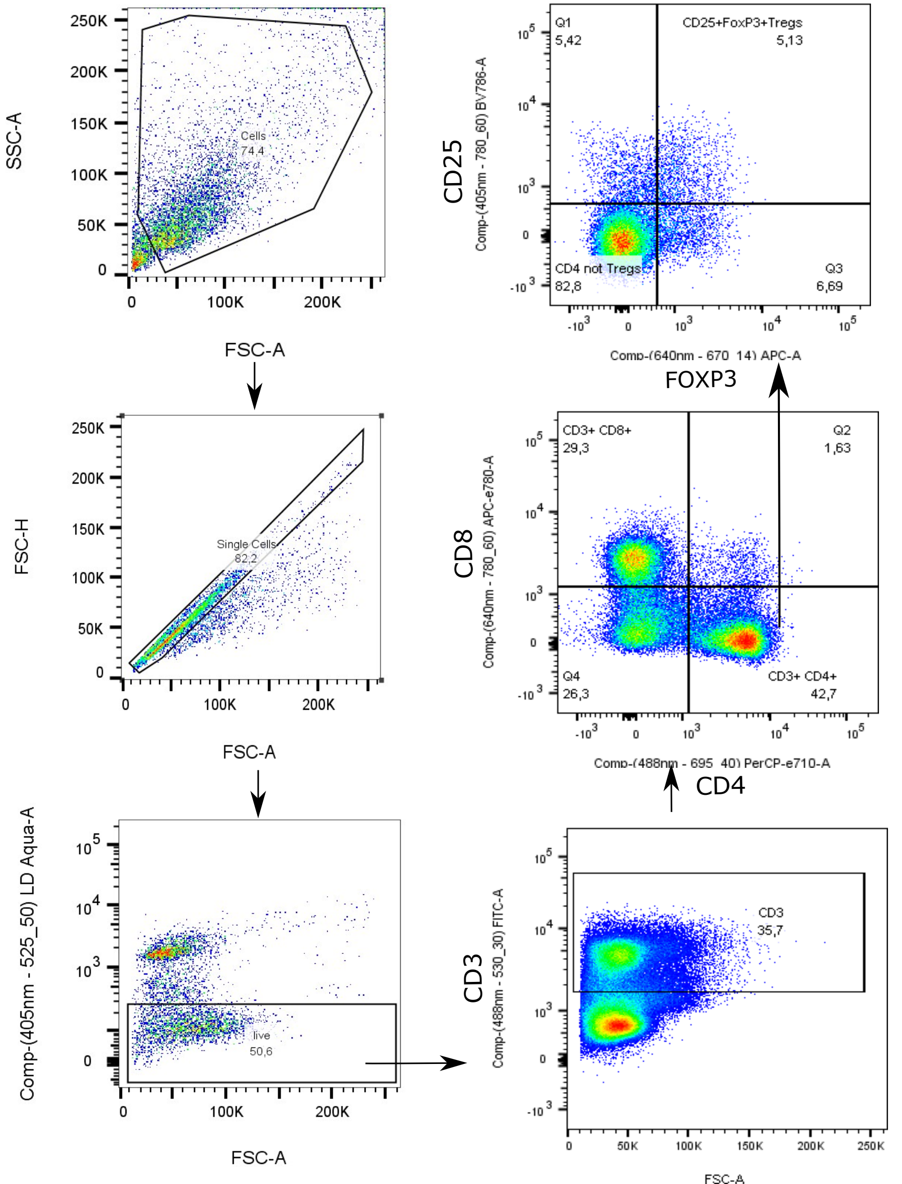

Supplement: Supplementary file 1 [file DataSheet1.PDF]
